# A Systematic Review Illustrates the Expanding Clinical and Molecular Landscape of Helsmoortel-Van der Aa Syndrome

**DOI:** 10.3390/brainsci16010004

**Published:** 2025-12-19

**Authors:** Lusine Harutyunyan, Claudio P. D’Incal, Anna C. Jansen, Marije Meuwissen, Anke Van Dijck, R. Frank Kooy

**Affiliations:** 1 Center of Medical Genetics, Department of Medicine and Health Sciences, University of Antwerp, 2000 Antwerp, Belgium; lusine.harutyunyan@uantwerpen.be (L.H.); claudio.dincal@uantwerpen.be (C.P.D.);; 2 Family Medicine and Population Health (FAMPOP), Department of Medicine and Health Sciences, University of Antwerp, 2000 Antwerp, Belgium; 3 Genetic Epilepsies and Neurodevelopmental Disorders Research Antwerp (GENERAte), Translational Neurosciences (TNW), Department of Medicine and Health Sciences, University Hospital Antwerp, University of Antwerp, 2000 Antwerp, Belgium; 4 Division of Pediatric Neurology, Departments of Pediatrics, Antwerp University Hospital, 2650 Edegem, Belgium

**Keywords:** Helsmoortel-Van der Aa syndrome, HVDAS, *ADNP*, autism, neurodevelopmental disorder, systematic review

## Abstract

**Background**: Helsmoortel-Van der Aa syndrome (HVDAS) is a rare multisystemic neurodevelopmental disorder caused by pathogenic variants in the *Activity-Dependent Neuroprotective Homeobox Protein (ADNP)* gene. Since the extensive clinical description of a cohort of 78 affected individuals in 2019, numerous reports described additional cases affected by the condition. However, no systematic synthesis of the clinical and molecular spectrum of these additional individuals has been conducted to date. **Methods**: In accordance with the PRISMA 2020 guidelines, we performed a systematic review of all publications describing individuals with genetically confirmed HVDAS. Clinical characteristics, comorbidities, and developmental milestones were systematically extracted to illustrate novel or underrecognized manifestations. **Results:** A total of 105 individuals reported across 34 publications were included. Of these, 66 were clinically and genetically evaluated, and 39 were analyzed only at the genetic level. Our analysis refines the phenotypic spectrum of HVDAS, including developmental delay, visual anomalies, and congenital heart defects. The additional literature also allows us to characterize in more detail the ophthalmological abnormalities, gait disturbances, and the cognitive profile of HVDAS. Advances in *ADNP* methylation profiling further enhance diagnostic precision and variant interpretation in this evolving neurodevelopmental syndrome. **Conclusions**: This systematic review provides a comprehensive synthesis of the clinical, genetic, and epigenetic landscape of HVDAS. It underscores the multisystemic nature of the disorder and the need for multidisciplinary management. The expanding phenotypic heterogeneity likely reflects both improved clinical recognition of the more subtle features and the tendency to prioritize publication of more complex or severely affected cases.

## 1. Introduction

Helsmoortel-Van der Aa syndrome (OMIM, 615873) is a rare, multisystemic neurodevelopmental disorder caused by heterozygous *de novo* variants in the *Activity-Dependent Neuroprotective Homeobox Protein (ADNP)* gene. *De novo ADNP* variants are one of the most frequent monogenetic causes of autism spectrum disorder (ASD), with an estimated global prevalence of 0.17% of ASD individuals [1,2,3]. *ADNP* encodes a putative transcription factor with differential interactions with proteins involved in chromatin remodeling [4]. It plays a crucial role in the neocortical neurogenesis [5] and contributes to neuronal differentiation through regulation of Wnt signaling [6]. The exact function of *ADNP* is still not fully determined; however, evidence points to a chromatin regulatory role of the protein [7].

Following its discovery in 2014 [1], we comprehensively described the clinical spectrum of Helsmoortel-Van der Aa syndrome (HVDAS) in a cohort of 78 individuals in 2019 [8]. The clinical presentation of this cohort is characterized by hypotonia, speech delays, global developmental delay, mild to severe intellectual disability (ID), ASD, attention deficit (hyperactivity) disorder (ADHD/ADD), and behavioral problems such as aggressive behavior, obsessive-compulsive behavior, mood disorder, high anxiety level, and self-injurious behavior. Dysmorphic facial features, such as a prominent forehead, a depressed nasal bridge, palpebral fissure anomalies and thin upper lip vermilion, and hand and foot abnormalities were also often reported. Systematic manifestations included frequent otitis media, feeding problems, including dysphagia to liquids, and gastrointestinal problems among which gastroesophageal reflux and constipation. Additional problems included sleep problems, seizures, cerebral abnormalities, congenital heart defect, ophthalmological problems, and recurring infections. Interestingly, the early eruption of primary teeth was reported in 81% of patients [9]. Currently, little knowledge exists about the long-term perspectives of the disorder, and no treatments are available [4,10]. Therapy primarily entails symptom alleviation through speech, occupational and physical therapy, nutritional support, and standard treatment of neuropsychiatric features, gastrointestinal, musculoskeletal and ophthalmological issues.

Since its initial description, a significant number of reports have described small cohorts or individual cases of affected individuals including clinical and molecular details [11,12,13,14,15,16,17,18,19,20,21,22,23,24,25,26,27,28,29,30,31,32,33,34,35,36]. However, no study has comprehensively compiled these cases. This systematic review includes all available additional publications on HVDAS and aims to thoroughly appraise the available clinical data and compare these features to the clinical spectrum described by Van Dijck et al. (2019) [8], to uncover new relevant clinical symptoms and provide a practical resource for clinicians and researchers working with this patient population. In addition, no previous systematic review incorporating methylation-based diagnostics and episignatures has been published.

## 2. Materials and Methods

A systematic review was performed based on the Preferred Reporting Items for Systematic Reviews and Meta-Analyses (PRISMA) 2020 guidelines [37]. This review was registered with the International Prospective Register of Systematic Reviews (PROSPERO) (CRD420251177699) on 27 October 2025.

The research question was structured using the PECO (Population, Exposure, Comparison, Outcomes) framework.

- Population (P): Individuals of any age and sex diagnosed with Helsmoortel-Van der Aa syndrome carrying a pathogenic *ADNP* variant.
- Exposure (E): Presence of a pathogenic or likely pathogenic *ADNP* variant identified through genetic testing in case reports or series.
- Comparison (C): The first HVDAS cohort study by Van Dijck et al. (2019) [8] consisting of 78 patients.
- Outcomes (O): A comprehensive compilation of clinical features, comorbidities and developmental milestones to identify both established and novel clinical features of HVDAS.

### 2.1. Search Strategy

We conducted a comprehensive literature search to identify eligible studies reporting Helsmoortel-Van der Aa syndrome cases, published in the PubMed and Web of Science databases. The final searches were completed on 25 September 2025. For both databases, the search terms were related to the population (individuals with HVDAS), comparison (clinical features), and outcome (clinical phenotypes, developmental milestones, comorbidities). We used the following MeSH and free-text terms: “Helsmoortel-Van der Aa syndrome”, “HVDAS”, “*ADNP*”, “*ADNP* AND case report”, “*ADNP* AND case”, “*ADNP* AND case study”, “*ADNP* AND case reports”.

### 2.2. Screening and Eligibility

Titles and abstracts were independently screened by two authors (L.H. and C.P.D.). Full-text screening was conducted by one reviewer (L.H.), with discussions involving C.P.D. and M.M. in cases of clinical uncertainty. Titles and abstracts were assessed according to the following inclusion criteria, which required that: (1) the publication type was a case report or case series, (2) published after the first description of HVDAS by Helsmoortel et al. (2014) [1], and (3) reporting individuals with confirmed *ADNP* variants and a clinical diagnosis of HVDAS. For full-text screening, we further excluded case reports describing (1) less than ten clinical features to ensure sufficient data for meaningful comparative analysis, (2) molecular or mechanistic research papers and reviews, (3) case reports with no genetically and clinically confirmed diagnosis of HVDAS, (4) non-retrievable publications, and (5) grey literature (conference abstracts, theses, etc.). Only papers available in the English language were considered. An exception was made for one Russian case report, which was translated using *DeepL Translator* and subsequently validated by a Russian-speaking clinician.

### 2.3. Data Extraction and Synthesis

Data extraction from the eligible publications was independently conducted by two authors (L.H. and C.P.D.). In case of uncertainty, full texts were reviewed and discussed with the other authors. Extracted study characteristics included author names, year of publication, country, study type, number of cases, and age and sex of the individuals. Clinical features of all reported cases were systematically compiled, following the structure of the first HVDAS cohort study [8]. Relevant clinical manifestations were selected and summarized in this review, with emphasis on the novel or underreported features. After excluding duplicates, a total of 66 individuals with HVDAS were included for systematic clinical analysis. All clinical and molecular data for each individual publication were grouped according to major physiological systems and domains, including: pre- and perinatal observations; morphological features; development and neurology; autistic features, behavior, and sleep; feeding and gastrointestinal problems; cerebral imaging, visual system; cardiovascular system; urogenital system; hand and foot abnormalities; growth and endocrine system; musculoskeletal system; auditory function; and additional problems. Data were tabulated using Microsoft Excel to enable consistent visualization and comparison across studies. Each feature was coded as present (+), absent (−), or not reported (empty cell), based on the available information in the original publication. To prevent duplication, overlapping patient reports across multiple studies were identified by comparing demographic and genetic variant information and were subsequently excluded from the clinical synthesis. Ultimately, from the remaining unique cases all clinical features were compiled into a single summary presenting the prevalence of each feature within the analyzed cohort. For all features, denominators reflect only the individuals for whom the feature was explicitly assessed or reported; cases with missing or unmentioned data were excluded from the denominator.

After this process, 66 unique individuals with HVDAS were retained for systematic clinical characterization. For reports that lacked sufficient phenotypic or developmental detail, only genetic variant information was extracted, resulting in a total of 105 individuals included in the molecular characterization dataset.

Given the heterogeneity of the data and the descriptive nature of the source material (primarily case reports and small series), a narrative synthesis approach was applied rather than quantitative meta-analysis. Emphasis was placed on identifying recurrent, refined, and novel clinical features relative to the previously established HVDAS phenotype.

### 2.4. Risk of Bias Assessment

Given that this review focuses on case reports and case series, the Joanna Briggs Institute (JBI) Critical Appraisal Checklists for both Case Reports and Case Series (2020) were applied to assess methodological quality and potential risk of bias [38]. These standardized tools provide a structured framework to evaluate the transparency and reliability of individual studies, addressing domains such as: (1) Patient demographics, assessing whether sufficient information is provided regarding patient background and medical history; (2) Diagnostic information, determining whether diagnostic methods and results, including genetic confirmation of an *ADNP* variant, are clearly reported; (3) Clinical features and management, evaluating the adequacy of clinical presentation, and treatment; and (4) Follow-up, assessing whether longitudinal outcomes are appropriately documented.

The use of both JBI tools ensured comprehensive quality appraisal across different study designs, enhancing consistency and reproducibility in bias assessment. The studies were assessed by one person (L.H.) and checked by C.P.D. Discrepancies were resolved by consensus or consultation with a third reviewer. This approach helped evaluate the risk of bias and the overall quality of the included studies.

## 3. Results

### 3.1. Study Characteristics

**Figure 1:**
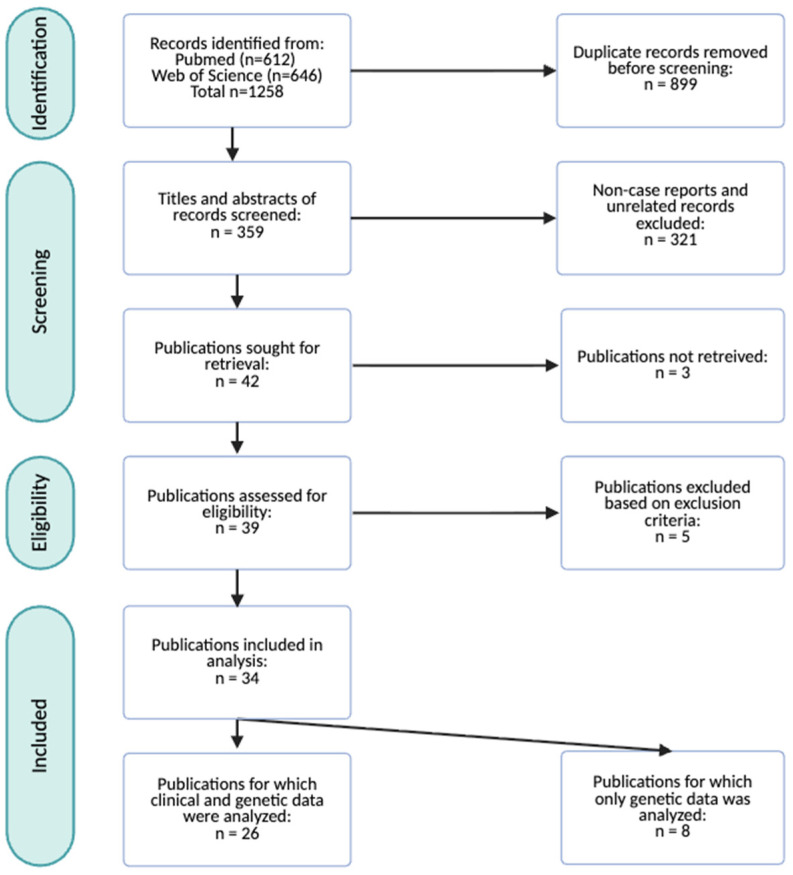
PRISMA flow diagram illustrating the record screening and study selection process. A total of 34 publications representing 105 individuals were included for this systematic review. Among these, 26 publications reporting on 66 individuals were analyzed to update the clinical characterization of HVDAS. Eight publications encompassing 39 cases did not contain sufficient clinical information, and for this reason only genetic information was extracted.

The study selection process followed the PRISMA 2020 guidelines and is summarized in the corresponding flow diagram (Figure 1). The initial literature search was conducted in PubMed and Web of Science, yielding a total of 1258 records. After removal of 899 duplicates, 359 unique records remained for screening.

During the title and abstract screening phase, 321 records were excluded as they did not meet the predefined inclusion criteria, and three reports were inaccessible. The full texts of the remaining 34 articles were assessed for eligibility. Five publications with insufficient current clinical data and without a genetically confirmed *ADNP* variant were excluded. After this stage, eight publications, corresponding to 39 cases, were excluded from clinical analysis because of insufficient clinical detail or absence of postnatal data. However, their genetic information was retained for molecular characterization of HVDAS.

Ultimately, 26 publications, comprising 66 individual cases, met the inclusion criteria and were included in this systematic review for qualitative synthesis and data extraction. These reports, together with eight published cohort studies and research papers (n = 39), form the basis for updated and expanded clinical and molecular characterization of HVDAS (n = 105) (Table 1). The raw dataset comprising all clinical data extracted from each included case is available in the Supplementary Materials.

### 3.2. Risk of Bias

Application of the JBI Critical Appraisal Checklists indicated that the overall methodological quality of the included case reports and case series was moderate to high (Table 1). Although, a few studies did not strictly meet the definition of a case report or case series, they were included in agreement with all authors as they contained sufficient clinical and demographic detail for extraction. Most case reports [11,12,13,14,15,16,17,18,19,20,21,22,23,24,25,26,27,28,29,33,34,35,36] clearly described patient demographics, clinical features, and genetic confirmation of an *ADNP*-variant. However, some domains showed variability in reporting quality. In particular, details on diagnostic methods and longitudinal follow-up were incomplete or absent in eight publications, but these differences did not substantially affect overall study quality. Criteria related to post-intervention clinical condition and adverse events were generally not applicable, as no disease-specific treatment for HVDAS currently exists. Case series [30,31,32] generally demonstrated higher methodological rigor than single case reports, although one lacked individual demographic data and two missed data on systematic follow-up.

### 3.3. Demographics

**Figure 2:**
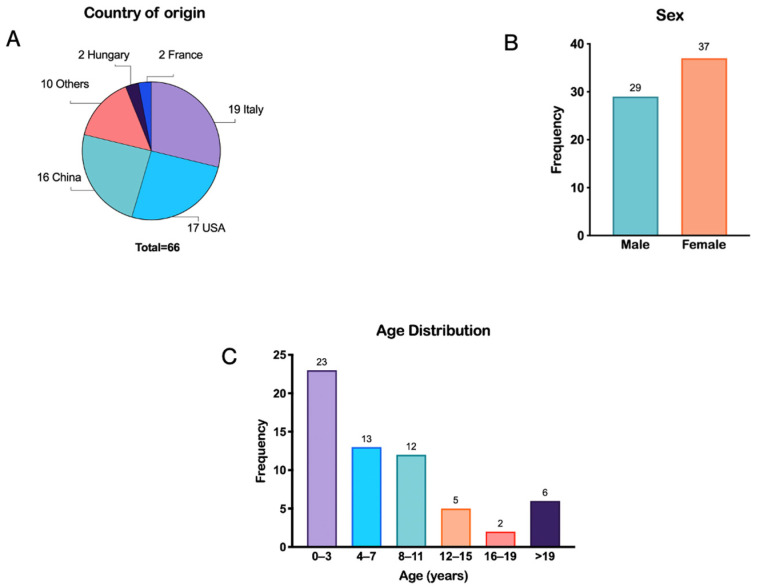
Demographic characteristics of the 66 reported individuals diagnosed with HVDAS. (**A**) Country of origin, (**B**) sex distribution, and (**C**) age distribution of the reported cases. The category “Others” includes Canada, Croatia, the Czech Republic, India, Israel, Japan, the Netherlands, Peru, Poland, and Saudi Arabia.

A total of 26 publications were included in this review, describing the clinical phenotype of 66 individuals with a pathogenic *ADNP* variant, originating from 15 different countries (Figure 2A) [11,12,13,14,15,16,17,18,19,20,21,22,23,24,25,26,27,28,29,30,31,32,33,34,35,36]. Among the individuals, 29 (44%) were male and 37 (56%) were female (Figure 2B). The mean age at evaluation was six years and three months, while the median age was nine years. Ages ranged from 8 months to 27 years (Figure 2C).

### 3.4. Clinical Presentation

Detailed clinical features extracted from literature are summarized in the additional Table A1. These data were merged with those of the original cohort study in the last column of the table, to provide updated prevalence estimates for each feature across the combined cohort consisting of 144 individuals (66 + 78).

#### 3.4.1. Pre- and Perinatal Observations

Mean birth weight of the total cohort was approximately 3000 g, mean birth length was 48.6 cm, and mean head circumference was 34.3 cm. Parental consanguinity was reported in two cases. Approximately 16% of individuals were born prematurely, consistent with the prevalence in the general population [47]. Three individuals presented with an arched palate, a feature not described in the initial cohort.

#### 3.4.2. Morphological Features

Craniofacial and morphological characteristics are among the most consistently re-ported features of HVDAS. Across published case reports and case series, a distinctive facial gestalt has emerged, characterized by a prominent forehead and high anterior hairline. Downslanted palpebral fissures were present in 50% of the investigated individuals. A bulbous and upturned nasal tip was reported in 55.3% of individuals, while a wide and depressed nasal bridge was observed in 55.9%. Epicanthus was reported in nine cases, often associated with blepharophimosis (congenital narrowing of the eyelid openings) and hypertelorism, each documented in five individuals.

Additional oromandibular findings, such as tented upper lip, microstomia, retrognathia, and crowded dentition, have emerged as novel, or previously underreported observations within the HVDAS clinical spectrum. Everted lower lip vermillion was present in 80.9% of the individuals in this cohort (21/26). One isolated case presented with a suspected diagnosis of molar-incisor hypomineralization, a qualitative enamel developmental defect of uncertain etiology [22]. Advanced eruption of teeth was described in 60.3% of cases. Although relatively rare, macrocephaly (n = 3) as well as microcephaly (n = 3) were observed, suggesting that deviations in head size, while uncommon, represent notable manifestations of the syndrome.

#### 3.4.3. Development and Neurology

Nearly all individuals in the total cohort presented with global developmental delay (98.5%, 130/132) and ID (98.2%, 108/110). Motor delay was present in all individuals with available data (95/98). Many children achieved independent walking at a younger age than initially reported. In the current cohort, 78.6% of the investigated cases (11/14) were nonverbal, while the prevalence estimate of the total cohort was 29.1% (25/86). Nonverbal individuals in our cohort had a mean age of eight years and seven months at the time of assessment. The prevalence of increased limb muscle tone was 6.5% (6/93) in the total cohort. Seizures were reported in 15.2% of the reported individuals. Nerve conduction studies revealed severe four-limb axonal motor polyneuropathy in one case [34], suggesting potential involvement of the peripheral nervous system in HVDAS.

#### 3.4.4. Gait Disturbances

A distinct observation was the presence of impaired gait in seven individuals (7/7) in the included case reports, suggesting a potential motor phenotype in HVDAS. In a recent systematic gait kinematics study in twelve individuals with HVDAS, reduced walking velocity, shortened step length, and increased step width were observed [48]. These observations may result from hypotonia and muscle weakness, both recognized hallmarks of the syndrome.

#### 3.4.5. Autistic Features, Behavior, and Sleep

Behavioral and neuropsychiatric features, including ASD (104/112; 92.9%), ADHD/ADD (35/69; 50.7%), and aggressive behavior (40/48; 83.3%), were common. Many children were treated with antipsychotic or stimulant medications to manage behavioral problems. Mood disorder and obsessive-compulsive disorder (OCD), were present, but less frequently reported in our cohort than in the original cohort [8]. Sleeping problems, based on parental reports, were present in 95.8% of individuals in our cohort and 73.1% in the total cohort.

#### 3.4.6. Gastrointestinal Problems

Feeding and gastrointestinal abnormalities were also reported, including gastroesophageal reflux (GERD) in 62% (49/79), constipation in 51.2% (42/82), and oral motor dysfunction in 46% (29/63). Based on the reported height and weight of the individuals at last clinical evaluation, 15% of the individuals (14/93) were obese.

#### 3.4.7. Cerebral Imaging

Neuroimaging findings in the current cohort included atypical cerebral white matter lesions which occurred in 6 out of 13 individuals with available MRI data (46.2%), delayed myelination (4/10), and cerebral atrophy (4/11). When considering the merged cohort, the prevalence of these findings was notably lower, suggesting that the higher proportions in the current dataset may be influenced by its smaller sample size. Imaging abnormalities generally remained mild and nonspecific (12/21), with their clinical significance yet to be determined.

#### 3.4.8. Visual System

Visual impairment constitutes a significant component of HVDAS. In total, hypermetropia was reported in 46.4% (39/84), strabismus in 54.5% (48/88), and ptosis in 32.1% (25/78). A longitudinal ophthalmological case study emphasized that visual deficits in some individuals stem not only from ocular anomalies but also from cerebral visual impairment (CVI), which occurred in 18 of the 40 individuals with detailed assessment (45%) in the merged cohort, indicating that both structural and functional visual abnormalities are common in HVDAS [17].

#### 3.4.9. Cardiovascular System

Cardiovascular anomalies predominantly comprised congenital structural heart defects rather than acquired cardiac disease. Among the 108 individuals with cardiac data available in the total cohort, 49 had a congenital structural anomaly, of which atrial septal defects were most common (21.4%, 18/84), followed by ventricular septal defect (11.6%, 10/86), and patent foramen ovale (9.6%, 8/83). Approximately 14.1% (13/92) of reported cardiac anomalies were unspecified in nature.

#### 3.4.10. Urogenital, Endocrine System and Growth

Cryptorchidism and small genitalia were observed in 41.5% (17/41) and 11.1% (9/81) of males in the total cohort, respectively. Short stature and thyroid hormone abnormalities remained as common features. Signs of precocious puberty, including early pubic hair growth and menarche, were reported in two females out of five investigated individuals in our cohort and in 33.3% (5/15) of the individuals in the total cohort. Additionally, a single case with polycystic ovaries and right solitary kidney was described [27].

#### 3.4.11. Hand and Foot Abnormalities and Musculoskeletal System

Abnormalities of the morphology of the extremities were common, including broad fingers, brachydactyly, clinodactyly, and short toes. Nail anomalies, such as thin, small, and/or hypoplastic nails, were observed in 30.2% of the investigated individuals in the merged cohort (19/63).

Chest wall deformities were occasionally reported. Trigonocephaly and plagiocephaly were documented in 6/77 (7.8%) and 10/77 (13%) total individuals, respectively.

#### 3.4.12. Ear, Nose, and Throat and Hearing

Among individuals who underwent formal auditory testing, hearing impairment was documented in 13.3% (11/83) of the total cases. Recurrent otitis media was reported in 63.6% of individuals (14/22) in the merged cohort, and obstructive sleep apnea occurred in 11% of individuals (10/91). Notably, no cases of narrow external hearing canal or the use of tympanostomy tubes were reported in this cohort.

#### 3.4.13. Additional Findings

Clinically reported umbilical and inguinal hernias occurred in 13.8% of individuals in the total cohort with available data (9/65). Recurrent infections were noticed in around half of individuals, encompassing upper respiratory and urinary tract infections, as well as isolated cases of osteomyelitis (distal fibula and calcaneus).

### 3.5. Prenatal Observations and Diagnosis

Prenatal diagnosis of HVDAS remains uncommon, as the disorder most often arises from a *de novo* heterozygous variant in *ADNP* and the clinical phenotype is often not evident in the prenatal setting. In most cases, the diagnosis is established postnatally, once developmental delays or behavioral problems become apparent. Prenatal identification typically occurs incidentally, when fetal anomalies detected on ultrasound prompt further genetic investigation. To date, only a limited number of prenatal cases have been reported [39,40,49]. Fetuses later confirmed to carry pathogenic *ADNP* variants have shown findings such as intrauterine growth restriction, skeletal malformations, congenital diaphragmatic hernia, ventriculomegaly, and cardiac anomalies. These findings are non-specific and overlap with a wide spectrum of neurodevelopmental disorders, limiting their diagnostic value for HVDAS. One prenatal case that led to pregnancy termination at 14 + 3 weeks presented with a complex phenotype, characterized by cerebral ventriculomegaly with choroid plexus abnormalities, bilateral absence of thumbs, left club-hand deformity, and a single atrioventricular canal [49]. Chromosomal microarray analysis revealed a *de novo* multigene deletion at 20q13.13.-q13.2 encompassing 38 genes among which Sal-like 4 (*SALL4*), Potassium voltage-gated Channel subfamily B member 1 (*KCNB1*), and *ADNP* are known to be associated with distinct genetic syndromes. The co-deletion of these dosage-sensitive genes precluded an unambiguous diagnosis of HVDAS, as the phenotype likely reflects combined or interacting pathogenic effects of *SALL4* and *ADNP*. Consequently, most HVDAS cases are recognized retrospectively, following molecular confirmation postnatally.

### 3.6. Mapping of Reported ADNP Variants in the Helsmoortel-Van der Aa Syndrome

**Figure 3:**
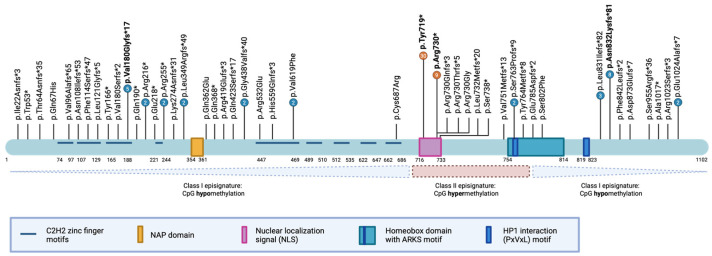
Schematic representation of the *ADNP* protein structure, functional domains, and reported variants. The *ADNP* protein contains defined domains, including nine C2H2 zinc finger motifs (light blue), the NAP octapeptide sequence (NAPVSIPQ; orange), a bipartite nuclear localization signal (NLS; pink), a DNA-binding homeobox domain harboring the alanine-arginine-lysine-serine (ARKS) motif (teal), and a PxVxL motif mediating the interaction with heterochromatin protein 1 (HP1, dark blue). Amino acid residue positions are indicated below the protein illustration. Vertical black lines denote the locations of reported variants in affected individuals, with four mutational hotspots highlighted in bold. The lower panel depicts the delineation of class 1 and class 2 episignatures, relative to the *ADNP* protein domains.

In total, we identified 105 individuals carrying pathogenic or likely pathogenic *ADNP* variants predicted to abolish normal protein function (Table A2). Across these cases, 48 unique protein-level variants were detected, encompassing 11 nonsense, 28 frameshift, seven missense variants, one intragenic inversion, and one splice-acceptor site variant (Figure 3). Consistent with previous reports [8,50,51], we observed recurrent mutational hotspots, including the most prevalent p.Tyr719* variant (30 individuals), the p.Arg730* variant (9 individuals), the p.Asn832Lysfs*81 variant (8 individuals), and the pVal180Glyfs*81 variant (5 individuals). Notably, we identified a previously unreported hotspot in the N-terminal region, the p.Val180Glyfs*17 variant, present in five unrelated individuals. Of the total variants, 92 were confirmed *de novo* (87.6%), 13 variants had unknown inheritance (12.4%), and none were inherited.

### 3.7. Methylation-Based Diagnosis for the Helsmoortel-Van der Aa Syndrome: Opposing Episignatures with Clinical Correlation

Initial genotype-phenotype analyses revealed that individuals carrying the p.Tyr719* variant achieved independent walking later and exhibited a higher pain threshold compared to carriers of other variants [8]. Moreover, the p.Tyr719* and neighboring variants were associated with an increased risk of blepharophimosis [12,15,18]. By contrast, variants mapping to the distal part of the nuclear localization signal (NLS), such as the p.Arg730* variant, were more frequently linked to ptosis and oromotor dysfunction [8].

A more robust genotype-phenotype relationship emerged following the discovery of dual methylation patterns, or episignatures, in peripheral blood from affected individuals [51,52]. Specifically, an episignature depends on the position of the variant within the *ADNP* gene: variants in the N- or C-terminal regions of the protein induce genome-wide CpG hypomethylation (class 1), whereas those within or adjacent to the NLS sequence and the proximal part of the homeobox domain result in CpG hypermethylation (class 2). These opposing episignature correlated modestly with the clinical presentation of affected individuals, reinforcing initial findings that the p.Tyr719* carriers exhibited more pronounced motor delay. Class 2 individuals additionally showed higher prevalence and severity of autism spectrum disorder [51,52]. Episignature testing may serve as a complementary diagnostic tool, alongside exome and genome sequencing, but its use is largely confined to research settings. It may aid, e.g., in reclassification of VUS when a matching episignature is identified. A negative episignature, however, does not exclude pathogenicity of a specific variant.

More recently, the application of artificial intelligence-based integrative tools such as PhenoScore, which combines facial image analysis, Human Phenotype Ontology (HPO) features, and methylation profiles, has strengthened these correlations. Using this approach, recurrent infections and gastrointestinal problems, including reflux, constipation, and feeding difficulties, were found to be two- to threefold more frequent among class 2 individuals compared to class 1 [50]. As mentioned above, individuals carrying the class 2 p.Tyr719* variant depicted blepharophimosis more frequently. Cases with blepharophimosis were demonstrated to have a distinct methylation signature similar to that of the Blepharophimosis with Intellectual disability syndrome (BIS), caused by *SMARCA2*-variants. This distinct methylation signature, termed BIS-HVDAS, which facilitates the molecular diagnosis of individuals with overlapping *ADNP*/*SMARCA2*-associated phenotypes [31].

## 4. Discussion

This systematic review provides the first comprehensive synthesis of the clinical spectrum associated with HVDAS. Our primary aim was to compile and critically appraise all reported clinical features linked to pathogenic *ADNP* variants and to identify emerging or previously unrecognized phenotypic traits relative to the first cohort by Van Dijck et al. (2019) [8].

### 4.1. Clinical Findings

#### 4.1.1. Confirmed Clinical Features

Hallmark features such as global developmental delay, ID, and autistic features remain consistently reported compared to the original cohort and within the merged cohort [8]. The reproducibility of craniofacial characteristics across independent studies reinforces their diagnostic relevance and underscores the value of facial pattern recognition in supporting clinical suspicion of HVDAS. The age at independent walking was slightly younger compared to the first cohort [8], though delayed motor development remained a defining feature. Similarly, emerging evidence suggests that congenital cardiovascular anomalies, although mild to moderate, represent a recurrent component of the syndrome, most commonly atrial and ventricular septal defects and patent foramen ovale.

Endocrine abnormalities, including thyroid and growth hormone dysregulation and early pubertal onset, remain common characteristics. Interestingly, *ADNP* mRNA expression fluctuates in the murine hypothalamus during the estrus cycle of mice, suggesting a possible role for *ADNP* in endocrine regulation [53]. These findings in mice warrant investigation into an endocrine regulatory role for *ADNP* in humans, though the underlying mechanisms remain to be elucidated.

Hearing impairment, either sensorineural or conductive, often linked to recurrent otitis media or structural anomalies, was also recurrently documented. In contrast, narrow external auditory canals, and the presence of tympanostomy tubes, which were reported in 87.5% and 73.3%, respectively, in the first cohort of Van Dijck et al. (2019) [8], were not reported in the present cohort. This discrepancy may reflect incomplete or age-dependent hearing examinations, differences in otitis media prevalence, or variations in reporting practices. These findings underscore the importance of comprehensive audiological evaluation at diagnosis and throughout follow-up.

#### 4.1.2. Refinement of Clinical Features

The proportion of individuals with ADHD/ADD and absent speech was substantially higher in the present cohort, which may suggest greater phenotypic severity. However, these differences could equally reflect improved clinical recognition or more targeted behavioral evaluation in recently reported individuals. The potential influence of ascertainment bias further underscores the importance of systematic and standardized assessments across future cohorts. Notably, when these features are examined within the merged cohort, the prevalence difference becomes far less pronounced and approximates the rates reported in the initial cohort, suggesting that larger, aggregated datasets provide a more balanced and representative view of the true phenotypic spectrum.

Neuroimaging data indicate that anomalies, such as delayed myelination, white matter changes, and mild cerebral atrophy were more frequent than initially reported [7]. These findings advocate for longitudinal neuroradiological follow-up, as the long-term developmental implications of these changes remain unclear.

The prevalence of obesity was surprisingly higher in this cohort. It remains unclear whether obesity represents a pathophysiological feature of HVDAS or arises secondary to associated neurodevelopmental dysfunction. Comparable observations have been made in other neurodevelopmental disorders, such as Fragile X syndrome [54,55].

Anomalies of the visual system were more frequently reported in this cohort, with ptosis emerging as the most recurrent feature. This observation may reflect improved recognition and reporting of subtle ocular manifestations. Nonetheless, when examining the merged cohort, prevalence estimates were notably lower and align more closely with those of the initial cohort, suggesting that the higher frequencies in our dataset likely reflect its smaller sample size rather than a true increase.

Similarly, the prevalence of cryptorchidism and small external genitalia appeared substantially higher in the present cohort compared with the 2019 cohort [8]. This apparent increase may be due to more detailed and systematically documented genital examination in more recent studies. However, consistent with the observations in the visual system, the proportions in the merged cohort were considerably lower, indicating that the strikingly high frequencies in our cohort are likely influenced by its limited sample size.

#### 4.1.3. Novel Clinical Features

Newly described features, particularly within the oromandibular region and visual systems, illustrate greater phenotypic variability than previously recognized. Abnormal gait patterns were increasingly documented, supporting motor coordination difficulties as a consistent yet variably expressed feature of the syndrome.

An additional, potentially underrecognized, manifestation of HVDAS involves skin anomalies. A detailed case report described a thin dermis, histologically confirmed, with periarticular hyperkeratosis and delayed wound healing [42]. Patient-derived keratinocytes showed reduced proliferation and premature differentiation, suggesting that *ADNP* dysfunction extends to skin homeostasis. Although these observations derive from a single case report, they raise the possibility that such dermatological features expand the multisystemic profile of HVDAS and may warrant considerations as accessible clinical markers in future research or surveillance.

Prenatal manifestations remain infrequently documented. When present, they include variable and often nonspecific structural anomalies detectable on fetal ultrasound or MRI. Therefore, the prenatal phenotype generally does not allow reliable classification of detected *ADNP* variants. In contrast, microdeletions encompassing *ADNP* and adjacent genes may produce more complex phenotypes, reflecting additive gene-dosage effects rather than isolated *ADNP* dysfunction [4].

### 4.2. Cognitive and Adaptive Profile

Most individuals with HVDAS exhibit moderate to severe ID [8], with full-scale IQ scores well below the normative range when assessed (mean verbal IQ = 32.64; mean nonverbal IQ = 31.18) [41]. Cognitive and adaptive functioning, assessed through instruments such as the Differential Ability Scales, 2nd Edition (DAS-II; n = 21) and the Vineland Adaptive Behavioral Scales (Vineland III; n = 4) [43,45,46], consistently reveal a profile of global developmental delay with marked deficits across all adaptive domains. Individuals typically function far below the age expectations, with slow progress and limited acquisition of new skills over time.

Language impairment, especially expressive deficits, represents a major clinical feature. Speech therapy may facilitate limited verbal progress, yet many individuals remain nonverbal, relying on gestures or sign language. Receptive language tends to be relatively better preserved but remains delayed. Over time, increasing reports of social withdrawal and aggression toward peers or adults have emerged [41]. Longitudinal data (n = 9) suggest that early developmental progress in motor, self-care, or communication domains often slows down with age, sometimes plateauing or declining during adolescence and adulthood [45].

Repetitive and restricted behaviors, including stereotyped motor movements, hand and finger mannerisms, licking, and prolonged visual inspection of objects are common features, regardless of whether individuals meet the full diagnostic criteria for ASD [8,41,56]. Sensory processing abnormalities are also frequent, affecting approximately 67% of individuals [8].

### 4.3. Clinical Recommendations

Given the multisystemic involvement of HVDAS, several domains warrant systematic evaluation and long-term follow-up [10]. At diagnosis, an extensive physical examination is recommended, followed by a set of baseline assessments recommended for all individuals and repeated regularly, including echocardiography, as mild congenital heart defects may otherwise remain undetected. A comprehensive audiological evaluation is recommended at diagnosis as well, as conductive and sensorineural hearing may fluctuate or progress, further impacting language acquisition and cognitive development, and evaluation of ophthalmologic functioning to identify ptosis and CVI in individuals with atypical visual function. Surveillance of growth parameters is recommended to evaluate both growth deficiency and obesity following initial diagnosis. Evaluation of thyroid function is recommended.

Physical therapy should be offered to address hypotonia, delayed gross motor milestones, and gait abnormalities, thereby enhancing functional mobility. Speech and language therapy should be initiated as soon as impaired language development in suspected and therapy is feasible, as early intervention could improve communicative outcomes and support social development [57].

Some conditional assessments, depending on the clinical presentation, should be performed. In individuals with short stature, evaluation of possible growth hormone deficiency should be considered

A comprehensive neurological evaluation is routinely recommended, while brain MRI is recommended in individuals presenting with atypical or concerning clinical signs. Additionally, an electroencephalogram (EEG) is advised when seizures are suspected, with appropriate anti-epileptic treatment initiated when indicated.

Ultrasound of the kidneys and bladder is also advised to assess for possible functional and/or structural anomalies.

### 4.4. Molecular Characterization of Helsmoortel-Van der Aa Syndrome

To date, 105 individuals harboring pathogenic *ADNP* variants have been reported, encompassing 48 unique alterations. The most recurrent alterations include p.Tyr719*, p.Arg730*, p.Asn832Lysfs*81, and pVal180Glyfs*17. Unexpectedly, seven individuals harbored missense variants, despite the relative absence of such variants in large-scale exome sequencing studies [58]. The pathogenic relevance of these missense variants therefore remains uncertain and will require experimental validation to confirm their functional impact.

Despite the growing number of reported cases, substantial phenotypic variability persists, suggesting that modifying genetic, epigenetic, and environmental factors contribute to the clinical heterogeneity of HVDAS. In individuals with microdeletions spanning the *ADNP* locus [49,59], haploinsufficiency was initially proposed as a potential pathogenic mechanism, given the loss of one functional copy of the gene. This underscores the importance of cautious interpretation in complex cases where overlapping genetic contributions may confound the clinical presentation.

To date, only limited evidence supports the association of deletions with HVDAS. However, the impact of deletions cannot be entirely excluded as the clinical features of these individuals are consistent with HVDAS. Although haploinsufficiency could plausibly explain the phenotype in an isolated case [59], more recent genotype-phenotype correlation studies challenge this hypothesis as the primary pathogenic mechanism in HVDAS due to the presence of two opposing episignatures [50].

Finally, emerging diagnostic approaches, such as *ADNP*-specific methylation profiling and facial recognition-based phenotyping offer potentially powerful complementary tools for variant interpretation and can aid the clinical diagnosis of HVDAS alongside exome or genome sequencing.

## 5. Conclusions

The broad phenotypic spectrum and pronounced interindividual variability across reported cases highlights the pleiotropic and multisystemic consequences of *ADNP* dysfunction. Beyond neurodevelopmental and behavioral manifestations, affected individuals may exhibit endocrine, urogenital, musculoskeletal, and cardiovascular involvement, warranting a multidisciplinary management approach. As the phenotypic spectrum continues to expand, it is increasingly evident that HVDAS rather is a continuum of neurodevelopmental and systemic features. Looking ahead, emerging diagnostic approaches, of *ADNP*-specific episignatures and AI-based facial phenotyping, hold considerable promise for refining future diagnostic criteria. These tools improve variant interpretation and thereby enhance early recognition of HVDAS.

Limitations of this study include variability in the accuracy of the case reporting and the inconsistent use of standardized phenotyping frameworks: here, absence of a feature in a given report does not necessarily indicate its absence in the individual but may reflect incomplete assessment or documentation. Denominators of individual features in Table A1 may vary and should therefore be interpreted with caution. In addition, although we took care to only include studies of adequate overall methodological quality, the risk-of-bias assessment revealed limitations inherent to case reports and case series. While most publications clearly described patient demographics, diagnostic confirmation, and clinical features, information on follow-up was often incomplete or absent. Criteria relating to post-intervention outcomes and adverse events were generally not applicable, as no disease-modifying treatments for HVDAS currently exist. These factors should be considered when interpreting the synthesized data.

Despite these limitations, a major strength of this systematic review lies in its comprehensive and duplicate-free literature synthesis. By integrating clinical, molecular, and epigenetic dimensions, this review provides the most up-to-date and detailed overview of the expanding landscape of HVDAS, offering valuable insights to guide future clinical practice, research priorities and diagnostic advancements.

## Figures and Tables

**Table 1:** Overview of all publications included for systematic review, presenting the number of unique cases, country of origin, study type, demographic information (sex and age) of individual cases and JBI risk of bias appraisal score for the publications included in the clinical and molecular data analysis. The first 26 publications in the table were analyzed for clinical and molecular characterization, while the rest were included only for molecular characterization. The appraisal checklist for case reports consists of eight items. The criteria regarding post-intervention clinical condition and adverse events were not applicable, because no disease-specific treatment for HVDAS is available at present, resulting in a maximum possible score of six. For case series the appraisal checklist consists of 10 items; however, the statistical analysis criterion was not applicable given the descriptive nature of the extracted data, yielding a maximum possible score of nine. ND = no data.

References	Number of Unique Cases	Country	Sex	Age (Years)	Risk-of-Bias Appraisal Score
Pescosolido et al., 2014 [11]	1	USA	F	6	6/6
Krajewska-Walasek et al., 2016 [12]	1	Poland	F	1.5	6/6
Gozes et al., 2017 [13]	1	USA	F	11	5/6
Li et al., 2017 [14]	1	Canada	M	12	6/6
Takenouchi et al., 2017 [15]	1	Japan	M	2.4	6/6
Alkhunaizi et al., 2018 [16]	1	Peru/China	M	5.8	6/6
Gale et al., 2018 [17]	1	USA	M	3.9	5/6
Pascolini et al., 2018 [18]	1	Italy	F	3.9	6/6
Levine et al., 2019 [19]	1	France/Poland	M	18	6/6
Kozhanova et al., 2020 [20]	1	Russia	F	2	5/6
Shillington et al., 2020 [21]	1	USA	F	1.6	6/6
Petruzzi et al., 2021 [22]	1	Italy	M	9	6/6
Szabó et al., 2022 [23]	2	Hungary	2 F	7; 5	6/6
Chen et al., 2023 [24]	1	China	F	4	6/6
Georget et al., 2023 [25]	1	France	F	3	4/6
Gozes & Shazman, 2023 [26]	1	Israel	M	1.3	6/6
Al-Enezi et al., 2024 [27]	1	Saudi Arabia	F	13	6/6
D’Incal et al., 2024a [28]	1	The Netherlands	F	5	4/6
D’Incal et al., 2024b [29]	1	Croatia	M	6	6/6
Sarli et al., 2024 [31]	12	USA	4 M; 8 F	ND	6/9
Ge et al., 2024 [30]	15	China	9 M; 6 F	range 1–7	7/9
Pascolini et al., 2024 [32]	15	Italy	6 M; 9 F	range 8–26	8/9
Holec & Gozes, 2025 [33]	1	Czech Republic	M	3.2	5/6
Scaccini et al., 2025 [34]	1	Italy	F	13	5/6
Benvenuto et al., 2025 [35]	1	Italy	F	24	6/6
D’Incal et al., 2025 [36]	1	USA	M	15	5/6
Asegaonkar et al., 2023 [39]	1	India	ND	foetus	/
Rosenblum et al., 2023 [40]	1	Belgium	F	0.10	/
Arnett et al., 2018 [41]	11	USA	8 M; 3 F	range 4–14	/
Mollinedo et al., 2019 [42]	1	Spain	F	11	/
Levine et al., 2022 [43]	4	Israel	3 M; 1 F	range 6.6–27	/
Gozes et al., 2024 [44]	1	Belgium	M	8	/
Levine et al., 2024 [45]	7	Israel	6 M; 1 F	ND	/
Neuhaus et al., 2024 [46]	13	USA	ND	age 3.9–15.6	/

## Data Availability

No new data were created or analyzed in this study. Data sharing is not applicable to this article.

## Supplementary Materials

The following supporting information can be downloaded at: https://www.mdpi.com/article/10.3390/brainsci16010004/s1, Table S1: The raw dataset consisting of a comprehensive compilation of all clinical data extracted from the 26 included publications describing individuals with genetically confirmed HVDAS.

## Abbreviations

The following abbreviations are used in this manuscript:

ADD	Attention Deficit Disorder
ADHD	Attention Hyperactivity Deficit Disorder
*ADNP*	Activity-Dependent Neuroprotective Homeobox Protein
ARKS	Alanine-arginine-lysine-serine motif
ASD	Autism Spectrum Disorder
BIS	Blepharophimosis with Intellectual disability Syndrome
CVI	Cerebral Visual Impairment
DPM1	Dolichyl-Phosphate Mannosyltransferase 1
EEG	Electroencephalogram
GERD	Gastroesophageal Reflux Disease
HP1	Heterochromatin protein 1
HPO	Human Phenotype Ontology
HVDAS	Helsmoortel-Van der Aa syndrome
ID	Intellectual Disability
JBI	Joanna Briggs Institute
KCNB1	Potassium voltage-gated Channel subfamily B member 1
MRI	Magnetic Resonance Imaging
NAPVSIPQ	NAP octapeptide sequence
NLS	Nuclear localization signal
PRISMA	Preferred Reporting Items for Systematic Reviews and Meta-Analyses
SALL4	Sal-like 4
SMARCA2	SWI/SNF Related BAF Chromatin Remodeling Complex Subunit ATPase 2
VUS	Variant of Uncertain Significance

## Appendix A

**Table A1:** Clinical features of reported cases with HVDAS in our cohort and our cohort merged with the initial cohort of Van Dijck et al. 2019 [8]. Denominators (Total N) represent the number of individuals for whom a given feature was assessed. The novel or underreported features are highlighted in bold. MRI = Magnetic Resonance Imaging.

		This Cohort (N = 66)	Total (N = 144)
Clinical Features		Cases n/Total N (%)	Cases n/Total N (%)
Pre- and Perinatal Observations			
consanguinity		2/17 (11.8)	0/0 (0)
premature		6/37 (16.2)	
birth weight (mean, g) *		3053.85	3060.65
birth length (mean, cm) **		48.6	49.2
head circumference at birth (mean, cm)		34	34.6
**arched palate**		**3/3 ^+^**	
**submucous cleft palate**		**1/4 ^+^**	
Morphological Features			
prominent forehead		11/17 (64.7)	53/81 (65.4)
high anterior hairline		17/25 (68)	50/91 (54.9)
wide, depressed nasal bridge		19/27 (70.4)	52/93 (55.9)
short nose		9/15 (60)	41/78 (52.6)
bulbous, upturned nasal tip		19/25 (76)	47/85 (55.3)
**blepharophimosis**		**5/5 ^+^**	
**hypertelorism**		**5/6 ^+^**	
**epicanthus**		**9/9 ^+^**	
downslanted palpebral fissures		21/22 (95.5)	41/82 (50)
narrow palpebral fissures		1/1 ^+^	15/59 (25.4)
long eyelashes		3/4 ^+^	13/64 (20.3)
abnormality of the pinna (ear malformations)		9/10 (90)	41/76 (53.9)
long philtrum		18/25 (72)	40/81 (49.4)
**short philtrum**		**5/5 ^+^**	
thin upper lip vermilion		26/34 (76.5)	71/98 (72.4)
**everted lower lip vermilion**		**21/26 (80.8)**	
**microstomia**		**2/2 ^+^**	
**tented upper lip vermilion**		**3/3 ^+^**	
**retrognathia**		**2/2 ^+^**	
advanced eruption of teeth		12/28 (42.9)	44/73 (60.3)
**crowded dentition**		**3/3 ^+^**	
widely spaced teeth		11/11 (100)	29/63 (46)
widely spaced nipples		4/18 (22.2)	15/72 (20.8)
**microcephaly**		**3/3 ^+^**	
**macrocephaly**		**3/3 ^+^**	
Development and Neurology			
intellectual disability		35/37 (94.6)	108/110 (98.2)
developmental delay		57/59 (96.6)	130/132 (98.5)
motor delay		24/24 (100)	95/98 (96.9)
age sitting independently (months, mean) ***		11	11.5
walking independently		10/12 (83.3)	76/88 (86.4)
age walking independently (months, mean) ****		24	27
speech delay		39/39 (100)	109/110 (99.1)
age first words (months, mean) *****		35	35.5
absent speech		11/14 (78.6)	25/86 (29.1)
developmental regression (loss of skills)		0/2 ^+^	12/61 (19.7)
delayed ability to toilet train		5/5 ^+^	48/58 (82.8)
seizures		3/25 (12)	15/99 (15.2)
hypotonia		25/36 (73.7)	79/105 (75.2)
hypertonia		3/15 (20)	6/93 (6.5)
Autistic Features, Behavior, and Sleep			
autistic behavior		40/43 (93.2)	104/112 (92.9)
attention deficit (hyperactivity) disorder		10/12 (83.3)	35/69 (50.7)
agressive behavior		20/24 (83.3)	40/48 (83.3)
obsessive-compulsive behavior		2/5 ^+^	18/30 (60)
mood disorder		3/7 ^+^	12/23 (52.2)
self-injurous behavior		7/19 (36.8)	9/29 (31)
reduced pain sensation		11/18 (61.1)	46/73 (63)
abnormal sensory processing		2/2 ^+^	30/44 (68.2)
sleep disturbance		23/24 (95.8)	68/93 (73.1)
Feeding and Gastrointestinal Problems			
gastroesophageal reflux		11/14 (78.6)	49/79 (62)
constipation		8/13 (61.5)	42/82 (51.2)
oral motor dysfunction		3/6 ^+^	29/63 (46)
hyperphagia		3/5 ^+^	25/58 (43.1)
dysphagia to liquids		2/5 ^+^	21/64 (32.8)
aspiration		2/5 ^+^	14/61 (23)
vomiting		3/5 ^+^	21/66 (31.8)
gastrostomy tube feeding		1/4 ^+^	9/67 (13.4)
obesity		9/26 (34.6)	14/93 (15.1)
unspecified gastrointestinal problems		10/15 (66.7)	
Cerebral Imaging			
abnormal cerebral white matter morphology		6/13 (46.2)	10/66 (15.2)
ventriculomegaly		7/14 (50)	22/65 (33.8)
delayed myelination		4/10 (40)	8/55 (14.5)
cortical dysplasia		0/7 ^+^	2/59 (3.4)
corpus callosum hypoplasia		2/8 ^+^	11/57 (19.3)
cerebral atrophy		4/11 (36.4)	12/56 (21.4)
unspecified abnormalities MRI		12/21 (57.1)	29/67 (43.3)
Visual System			
hypermetropia		14/22 (63.6)	39/84 (46.4)
strabismus		17/25 (68)	48/88 (54.5)
myopia		2/4 ^+^	7/67 (10.4)
**astigmatism**		**9/9 ^+^**	
cerebral visual impairment		4/6 ^+^	18/40 (45)
**ectropion**		**1/1 ^+^**	
coloboma		3/5 ^+^	7/77 (9.1)
nystagmus		2/5 ^+^	11/82 (13.4)
ptosis		10/16 (62.5)	25/78 (32.1)
unspecified abnormalities of eye		10/15 (66.7)	
Cardiovascular System			
atrial septal defect		7/15 (46.7)	18/84 (21.4)
patent ductus arteriosus		1/10 (10)	7/79 (8.9)
patent foramen ovale		4/14 (28.6)	8/83 (9.6)
mitral valve prolapse		2/11 (18.2)	6/80 (7.5)
ventricular septal defect		7/17 (41.2)	10/86 (11.6)
tetralogy of Fallot		0/8 ^+^	1/77 (1.3)
unspecified cardiovascular abnormalities		7/23 (30.4)	13/92 (14.1)
Urogenital System			
cryptorchidism		5/6 ^+^	17/41 (41.5)
small external genitalia		5/7 ^+^	9/81 (11.1)
renal anomalies		1/5 ^+^	7/53 (13.2)
unspecified urogenital abnormalities		5/15 (33.3)	
Hand- and Foot Abnormalities			
abnormal finger morphology		22/33 (66.7)	53/100 (53)
single palmar crease		1/3 ^+^	8/68 (11.8)
sandal gap between toes		9/9 (100)	20/65 (30.8)
abnormality of toes		14/15 (93.3)	21/80 (26.3)
abnormality of nails		5/7 ^+^	19/63 (30.2)
unspecified hand and foot deformities		16/25 (64)	
Growth and Endocrine System			
short stature		11/30 (36.7)	27/99 (27.3)
hormone deficiency	thyroid hormone problem	3/7 *	10/53 (18.9)
	GH deficiency	1/5 ^+^	6/51 (11.8)
early puberty		2/5 ^+^	5/15 (33.3)
Musculoskeletal System			
joint hypermobility		4/8 ^+^	27/69 (39.1)
abnormality of the hip		0/2 ^+^	4/55 (7.3)
scoliosis		1/3 ^+^	12/67 (17.9)
pectus excavatum		2/4 ^+^	10/58 (17.2)
pectus carinatum		1/3 ^+^	4/57 (7)
abnormal skull shape	trigonocephaly	4/5 ^+^	6/77 (7.8)
	plagiocephaly	4/5 ^+^	10/77 (13)
	brachycephaly	2/4 ^+^	5/76 (6.6)
thoracic hypoplasia		0/2 ^+^	1/56 (1.8)
unspecified abnormalities of musculoskeletal system		2/15 (13.3)	
Ear-Nose-Throat and Hearing			
narrow external auditory canal		0/7 ^+^	7/15 (46.7)
recurrent otitis media		2/8 ^+^	14/22 (63.6)
tympanostomy tube placement		0/6 ^+^	11/21 (52.4)
hearing impairment		4/23 (17.4)	11/83 (13.3)
obstructive sleep apnea		5/15 (33.3)	10/91 (11)
Additional Problems			
**ataxic gait**		**7/7 ^+^**	
umbilical/inguinal hernia		4/6 ^+^	9/65 (13.8)
recurrent infections	upper respiratory tract infection, urinary tract infection, osteomyelitis	14/28 (50)	49/97 (50.5)

*: total n = 19; n = 63. **: total n = 15, n = 45. ***: total n = 11; n = 58. ****: total n = 14; n = 64. *****: total n = 7; n = 49. ^+^ interpret number of individuals with caution; absence of feature may not have been assessed in the cohort.

## Appendix B

**Table A2:** Overview of all reported *ADNP* variants in literature. NK = not known.

Codon Variant	Protein Variant	Frequency Variant	Variant Type	References
c.2156dupA c.2157C>A c.2157C>G c.2157delC c.2157insT	p.Tyr719*	30	nonsense	Pescosolido et al., 2014 [11]; Gozes et al., 2017 [13]; Takenouchi et al., 2017 [15]; Arnett et al., 2018 [41]; Gale et al., 2018 [17]; Pascolini et al., 2018 [18]; Mollinedo et al., 2019 [42]; Sarli et al., 2024 [31]; Ge et al., 2024 [30]; Neuhaus et al., 2024 [46]; Pascolini et al., 2024 [32]; Scaccini et al., 2025 [34]
c.2188C>T	p.Arg730*	9	nonsense	Krajewska et al., 2016 [12]; Pascolini et al., 2018 [18]; Sarli et al., 2024 [31]; Ge et al., 2024 [30]; Levine et al., 2022 [43]; Pascolini et al., 2024 [32]
c.2496_2499delTAAA	p.Asn832Lysfs*81	8	frameshift	Arnett et al., 2018 [41]; Shillington et al., 2020 [21]; Pascolini et al., 2024 [32]; Levine et al., 2024 [45]; Holec and Gozes, 2025 [33]
c.539_542delTTAG	p.Val180Glyfs*17	5	frameshift	Levine et al., 2022 [43]; Szabó et al., 2022 [23]; Neuhaus et al., 2024 [46]
c.2491_2494del TTAA	p.Leu831Ilefs*82	3	frameshift	Li et al., 2017 [14]; Ge et al., 2024 [30]; Pascolini et al., 2024 [32]
c.1855G>T	p.Val619Phe	2	missense	Neuhaus et al., 2024 [46]
c.646C>T	p.Arg216*	2	nonsense	Levine et al., 2022 [43]; Levine et al., 2024 [45]
c.673C>T	p.Arg225*	2	nonsense	Ge et al., 2024 [30]; Pascolini et al., 2024 [32]
c.1255delA	p.Arg419Glufs*3	2	frameshift	Levine et al., 2022 [43]; Levine et al., 2024 [45]
c.3070delG	p.Glu1024Alafs*7	2	frameshift	Levine et al., 2024 [45]
c.2287delT	p.Ser763Profs*9	2	frameshift	Arnett et al., 2018 [41]; Sarli et al., 2024 [31]
c.1046_1047delTG	p.Leu349Argfs*49	2	frameshift	D’Incal et al., 2025 [36]
c.568C>T	p.Gln190*	1	nonsense	Chen et al., 2023 [24]
c.-89-3923_201 + 2793 inv	p.[?];[(=)]	1	intragenic inversion	Georget et al., 2023 [25]
c.[-5-1_-4del];[=]	p.[?];[(=)]	1	splice-acceptor site	D’Incal et al., 2024a [28]
c.1102C>T	p.Gln368*	1	nonsense	Arnett et al., 2018 [41]
c.1265dup	p.Gln423Serfs*17	1	frameshift	Al-Enezi et al., 2024 [27]
c.1313delG	p.Gly438Valfs*40	1	frameshift	Neuhaus et al., 2024 [46]
c.158G>A	p.Trp53*	1	nonsense	Petruzzi et al., 2021 [22]
c.1595G>A	p.Arg532Glu	1	missense	Neuhaus et al., 2024 [46]
c.1676dupA	p.His559Glnfs*3	1	frameshift	D’Incal et al., 2024b [29]
c.190dupA	p.Thr64Asnfs*35	1	frameshift	Arnett et al., 2018 [41]
c.201G>C	p.Gln67His	1	missense	Neuhaus et al., 2024 [46]
c.2189del	p.Arg730Glnfs*3	1	frameshift	Pascolini et al., 2024 [32]
c.2194_2197delTTAG	p.Leu732Metfs*20	1	frameshift	Neuhaus et al., 2024 [46]
c.2213C>A	p.Ser738*	1	nonsense	Szabó et al., 2022 [23]
c.2222dupT	p.Phe842Leufs*2	1	frameshift	Pascolini et al., 2024 [32]
c.2250_2274del	p.Val751Metfs*13	1	frameshift	Arnett et al., 2018 [41]
c.2289delC	p.Tyr764Metfs*8	1	frameshift	Ge et al., 2024 [30]
c.2355_2356del AA	p.Glu785Aspfs*2	1	frameshift	Ge et al., 2024 [30]
c.2405C>T	p.Ser802Phe	1	missense	Benvenuto et al., 2025 [35]
c.2619_2620delCA	p.Asp873Glufs*7	1	frameshift	Asegaonkar et al., 2023 [39]
c.287del	p.Val96Alafs*65	1	frameshift	Arnett et al., 2018 [41]
c.3047insT	p.Ala1017*	1	nonsense	Kozhanova et al., 2020 [20]
c.3069_3072del	p.Arg1023Serfs*3	1	frameshift	Rosenblum et al., 2023 [40]
c.319del	p.Asn108Ilefs*53	1	frameshift	Pascolini et al., 2024 [32]
c.340delG	p.Phe114Serfs*47	1	frameshift	Arnett et al., 2018 [41]
c.361_362delTT	p.Leu121Glyfs*5	1	frameshift	Neuhaus et al., 2024 [46]
c.498_499del	p.Tyr166*	1	nonsense	Ge et al., 2024 [30]
c.537dupA	p.Val180Serfs*2	1	frameshift	Levine et al., 2019 [19]
c.64dupA	p.Ile22Asnfs*3	1	frameshift	Ge et al., 2024 [30]
c.651_655del	p.Glu218*	1	nonsense	Alkhunaizi et al., 2018 [16]
c.819delC	p.Lys274Asnfs*31	1	frameshift	Arnett et al., 2018 [41]
c.1084C>G	p.Gln362Glu	1	missense	Gozes and Shazman, 2023 [26]
c.2187dupA	p.Arg730Thrfs∗5	1	frameshift	Gozes et al., 2024 [44]
c.2865_2868del	p.Ser955Argfs*36	1	frameshift	Levine et al., 2024 [45]
c.2059T>C	p.Cys687Arg	1	missense	Ge et al., 2024 [30]
c.2188C>G	p.Arg730Gly	1	missense	Ge et al., 2024 [30]
